# Restoring productivity of degraded mined soils using legume leaf residues as organic amendments

**DOI:** 10.1038/s41598-026-41755-1

**Published:** 2026-03-06

**Authors:** Enoch Opoku, Beloved M. Dzomeku, John Opata, Adam M. Adam, Frank Rasche

**Affiliations:** 1 Department of Agronomy in the Tropics, Institute of Tropical Agricultural Sciences (Hans-Ruthenberg-Institute), University of Hohenheim, Stuttgart, Germany; 2 Council for Scientific and Industrial Research, Crops Research Institute, Kumasi, Ghana; 3 University of Environment and Sustainable Development, Somanya, Ghana; 4 International Institute of Tropical Agriculture, Nairobi, Kenya

**Keywords:** Subsoil, Legumes, Soil amendments, Plant residues, Organic carbon and nitrogen, Restoration, Environmental sciences, Plant sciences

## Abstract

Scarcity and cost of topsoil and conventional materials make legume residues a promising alternative for reclaiming land degraded by opencast mining. This study examined plant residues by evaluating the effect of their quality (C:N ratio, lignin, polyphenols) on soil organic carbon (SOC), total soil nitrogen (TN) and crop (maize, cowpea) performance. A 12-month pot experimentation in Kumasi (Ghana) tested leafy residues of *Leucaena leucocephala*, *Gliricidia sepium*, *Mucuna pruriens*, *Pueraria phaseoloides*, and *Panicum maximum* (control), applied at four rates (0, 10, 20, 30 t ha⁻¹ dry weight) using a factorial randomized complete block design with four replications. Legumes had higher N content (≈ 3.5% by *Leucaena*) and higher quality indices (highest PRQI of 4.7 by *Gliricidia*), but lower lignin content than *Panicum.* SOC and TN gains were high (≈ 500% SOC and > 800% TN gains by *Mucuna* and *Leucaena*, respectively), explained by low initial concentrations of the substrate. Although mineralization was slow, plant quality indicators correlated with SOC and TN. Maize, other than cowpea, responded well to residues, with *Leucaena* (30 t ha⁻¹) yielding the highest dry matter (≈ 4.2 t ha⁻¹). Legume residues provide a sustainable solution for reclaiming opencast-mined land, showing subsoil improvement potential that requires field trials to validate pot-scale findings.

**Supplementary Information:**

The online version contains supplementary material available at 10.1038/s41598-026-41755-1.

## Introduction

In recent years, both large- and small-scale opencast gold mining have increased in several tropical countries, resulting in landscape degradation, deforestation, and intensified soil erosion^1^. In Ghana, this broader trend is reflected most prominently in the rapid expansion of illegal small-scale mining, locally referred to as *galamsey*. The rise of such mining activities has caused extensive destruction of productive farmland, rich forest vegetation, and key watersheds of high socioeconomic importance^2–5^. It was reported that approximately 47,000 ha of vegetation were converted for gold mining in Southwestern Ghana, with an annual average conversion rate of 2,600 ha^6^. Surface gold mining was also identified as the primary driver of land degradation in Ghana, contributing to the loss of approximately 13% of forested land^7^.

The lack of effective topsoil management and physical reconstruction often results in the complete loss or replacement of original topsoil with low quality subsoil^7,8^. This deficiency of quality topsoil remains a major constraint in the reclamation of mined lands^9^. Low organic matter content and insufficient concentrations of dissolved minerals in the subsoil render it incapable of supporting agricultural or forestry activities^10^.

A constant phenomenon of mining is the detrimental effects on important soil biological processes, soil physical and chemical properties (texture, bulk density, pH, soil organic carbon (SOC), nutrient availability)^4,5,11^. For example in Ghana, lower SOC (0.74%) and nitrogen (0.04%) contents in degraded gold mining soils were reported^12^. Soil microbial populations, essential for ecosystem functions, are also substantially reduced due to vegetation removal, topsoil stockpiling, and mixing with subsoil materials^13^. A 5 to 8 fold reduction in microbial populations in mine dumps was reported^14^.

Common reclamation practice is application of organic amendments such as municipal solid waste, animal manure, compost and alike to improve soil quality^10,15^. These can boost soil organic matter, with compost applications of 18–146 t ha^− 1^ increasing it by 6–163%, depending on material quality and quantity^16^. However, municipal materials may pose risks of pathogens and contaminants, while limited availability and high transport costs restrict their use in remote mining areas. Alternatively, certain perennial and annual legumes produce high-quality leaf materials rich in nitrogen (N) and other essential nutrients, offering a readily decomposable organic input that can significantly enhance soil structure, fertility, and biological activity, making them particularly effective for restoring degraded soils. Species such as *Leucaena leucocephala*,* Gliricidia sepium*, *Pueraria phaseoloides* and *Mucuna pruriens*, commonly found across Ghana, have demonstrated benefits for soil health through increased SOC, improved nutrient cycling, and biological nitrogen fixation (BNF)^17–19^. In contrast to conventional amendments, which have received considerable research attention, legume residues are locally available, cleaner, and more sustainable resources that can provide both in-situ and ex-situ benefits. However, their potential for rehabilitation of severely degraded soils remains largely unexplored. No available studies to date have systematically evaluated their use as organic amendments in subsoil environments, particularly in relation to optimal residue quality and quantity, and their effects on SOC and N dynamics in severely degraded soils. Recent studies in Ghana explored the use of animal manure and compost for soil restoration, although their utilization is constrained by several logistical and agronomic challenges previously discussed^20,21^. Furthermore, our study focused on legume residues derived from plants grown directly on degraded mined subsoils, reflecting the actual conditions under which large-scale biomass production is likely to occur unlike related studies on certain plant residues usually derived from fertile soils. Given the originality and large knowledge gaps in exploring legume leaf materials as organic amendment for mined land restoration, we propose that controlled pot experiments constitute the necessary first step before advancing to field-scale trials, as they enable the identification of key management and adaption strategies.

In this article, we hypothesized that the application of legume plant residues will increase SOC and N contents and enhance the productivity of subsoil substrates. The chemical composition (quality) and application rate (quantity) of these residues are expected to interact differently across species, influencing their amendment potential. Furthermore, it is anticipated that legume-based amendments will exert greater positive effects on subsoil SOC, N dynamics and crop performance compared to a non-legume control (*Panicum maximum*), commonly found on degraded sites and managed as weeds on farmlands which could lead to sustainable management of this species if beneficial outcomes are observed through this study. The specific objectives were to: (1) assess the chemical composition of selected legume and non-legume aboveground residues and identify key quality indicators mediating soil C and N dynamics in the subsoil of a degraded mined site; (2) quantify the contribution of these plant amendments to carbon and nitrogen stocks and evaluate their potential in regulating the subsoil C:N ratio toward optimal ranges for improved soil productivity; and (3) determine the threshold application rates of selected plant residues required to enhance the productivity of subsoil substrates in degraded mined environments.

## Materials and methods

### Experimental design

The pot experiment was conducted at the Council for Scientific and Industrial Research-Crops Research Institute (CSIR-CRI) located in Kumasi (Ghana) between June 2018 and June 2019 (including preparatory periods between crop cycles). The experiment was set up under a rain-out shelter with transparent polyurethane sheets as roof cover. Subsoil samples as substrate and fresh plant residues (leaf materials) as organic amendments were collected from two administrative (the Ashanti and Eastern) regions of Ghana. The soil samples, which were mixed together thoroughly to achieve high homogeneity prior to filling pots, were collected from abandoned small-scale mining site at Anumso (within latitude 6°44’16"N and longitude 1°13’58"W) in the Bosome-Freho District in the Ashanti Region. Residues from selected legume species and a dominant grass species (regarded as weed) as control were collected from a revegetated mined site at Asikam (within latitude 6°11’37"N and longitude 0°32’19"W) in the Abuakwa South Municipal District in the Eastern Region.

The pot experiment was a factorial experiment arranged in randomized complete block design with seven residue treatments (fresh plant residue as organic amendments) at different rate of application and replicated four times. The plant residues investigated were: (1) *Leucaena leucocephala*; (2) *Gliricidia sepium*; (3) *Mucuna pruriens*; (4) *Pueraria phaseoloides*; (5) *L. leucocephala* + *G. sepium* combined; (6) *M. pruriens* + *P. phaseoloides* combined; (7) *Panicum maximum* as a non-legume (weed) control. Combined residue treatments (1:1 to obtain equivalent quantities on dry matter basis of individual residues) were included to assess potential additive effects, improved nutrient release synchrony, and sustained nutrient availability, owing to differences in residue quality and decomposition rates. Four residue application rates were tested: 0, 0.1, 0.2, and 0.3 kg per kg of soil (equivalent to approximately 0, 30, 60, and 90 g dry weight, respectively). Based on the surface area of the pot substrate (0.031 m²), these rates correspond to field-equivalent applications of approximately 0, 10, 20, and 30 t ha^− 1^ (dry weight). A non-amended substrate served as control. In view of this, the experiment comprised a total of 112 experimental units (7 residue treatments × 4 residue application rates × 4 replicates). Maize (*Zea mays*; Omankwa variety) and cowpea (*Vigna unguiculata*; Agenkwa erect variety) obtained from cereals and grain division of CSIR-CRI were used as test crops in rotation to evaluate the productivity enhancement potential of the selected plant residues. Following one-time residue incorporation into the subsoil substrates within the pots at the beginning of the three sequential trials, treatments were irrigated manually using watering can to facilitate decomposition and nutrient mineralization and continued twice a week after sowing of test crops. Four weeks after amendment application, the first maize crop was sown. Upon completion of the maize trial (12 weeks), cowpea was sown (allowed to grow for a period of 8 weeks), followed by a second maize trial (12 weeks). In each trial, seedlings were thinned to two plants per pot after germination to ensure uniformity in plant density.

### Sample collection

Before initiating the pot experiment, a composite substrate sample was collected from the degraded mined site at a depth of 0–15 cm for baseline laboratory analysis (i.e. SOC and TN). Following the harvest of aboveground maize biomass in the first trial, two composite substrate samples were collected from each treatment to evaluate carbon and nitrogen mineralization dynamics in response to the plant residue treatments. In addition, two composite samples of each plant residue type were collected prior to incorporation for chemical characterization.

### Plant and soil sample analysis

Substrate samples collected before the pot experiment and after the first maize trial were air-dried, milled and sieved to 2 mm, then analyzed for SOC and TN. Soil OC content was analyzed using Walkley and Black method^22^. Total N was analyzed using Kjeldahl digestion and distillation procedure described by^23^.

Composite samples of each plant residue, comprising leaves, vines, and twigs with attached tree legume leaves approximately 50 cm from the tip, were analyzed in the laboratory to determine their lignin (LG), polyphenol (PP), organic carbon (OC), TN, available phosphorus (P), and potassium (K) contents. Lignin was quantified using the Acid Detergent Fiber (ADF) method developed by^24^ while PP content was measured using the Folin-Denis method as described by^25^. Organic C was determined by dry combustion using elemental analyzer, and TN by the Kjeldahl digestion and distillation method. Available P was analyzed using the vanadium phosphomolybdate method, and potassium was measured by atomic absorption spectrophotometry. All plant and soil sample analysis were conducted in the laboratories at the Soil Research Institute of the Council for Scientific and Industrial Research in Kumasi, Ghana.

To predict the quality of tested plant residues, the Plant Residue Quality Index (PRQI) was applied to evaluate their impact on soil and crops using “equation (1)”^26^. This index is based on the relative contributions of the C:N ratio, LG content (%), and PP content (%) to PRQI.

1 $$\mathrm{P}\mathrm{R}\mathrm{Q}\mathrm{I}=\left(\frac{1}{\mathrm{a}\mathrm{C}:\mathrm{N}+\mathrm{b}\mathrm{L}\mathrm{i}\mathrm{g}\mathrm{n}\mathrm{i}\mathrm{n}+\mathrm{c}\mathrm{P}\mathrm{o}\mathrm{l}\mathrm{y}\mathrm{p}\mathrm{h}\mathrm{e}\mathrm{n}\mathrm{o}\mathrm{l}\mathrm{s}}\right)\mathrm{x}100$$

where a, b, and c are coefficients of relative contributions of the above-mentioned residue indicators to residue quality. In this experiment, we used the parameters proposed by^26^ based on series of experiments: *a* = 0.423, *b* = 0.439 and *c* = 0.138.

The organic C and N mineralization rate constants $\it \left({\mathrm{k}}_{\mathrm{C}\mathrm{o}\mathrm{r}\mathrm{N}}\right)$ were deduced using “equation (2)” as illustrated below which was proposed by^27,28^:

2 $${\mathrm{k}}_{\mathrm{C}\mathrm{o}\mathrm{r}\mathrm{N}}=\mathrm{l}\mathrm{n}\left(\raisebox{1ex}{$\mathrm{x}$}\!\left/\!\raisebox{-1ex}{${\mathrm{x}}_{0}$}\right.\right)/\mathrm{t}$$

where x_0_ and x is amount of organic C and N at time zero and time t in days.

### Plant growth and performance data collection

For the two maize trials, data on plant height and Soil Plant Analysis Development (SPAD) meter readings were collected every two weeks after sowing. Twelve weeks after planting, aboveground biomass was harvested to measure both fresh and dry matter accumulation. Due to the shorter growth period of cowpea, data on plant height and SPAD readings were collected starting one week after sowing and then weekly until five weeks after sowing. At maturity, pods were harvested to assess treatment effects on cowpea grain yield.

### Statistical data analysis

Data collected were analyzed using JMP Pro 18 statistical software. All datasets were checked for normality prior to analysis. Maize plant height and SPAD (*n* = 8 for each crop sequence) and aboveground dry matter (*n* = 4 for each crop sequence) were analyzed using the mixed model procedure with replication (block) and Pot ID as the random effects while crop sequence (cycle), residue type, quantity applied and respective interactions were the fixed effects. Although cowpea was part of the sequential trials, it was treated separately due to the different growth characteristic and yield parameter collected. Therefore, data on cowpea (height, SPAD and grain yield) with n values similar to maize were analyzed using two-way analysis of variance (ANOVA). One way ANOVA was used to assess significant differences between plant residues chemical composition (*n* = 2). An alpha (α) level of 0.05 was used for all statistical tests. Where significant differences were detected between treatment means, Tukey’s Honest Significant Difference (HSD) post-hoc test was used to separate treatment means. Additionally, Spearman’s nonparametric correlation analysis was conducted to assess the relationships between the chemical composition of plant residues, SOC and TN contents following organic amendment and maize cultivation, and the performance of test crops. Graphical representations of the results were generated using the Matplotlib package in Python.

## Results

### Chemical composition of selected plant residues and residue quality assessment

The N content of all legume plant residues differed from *Panicum* (*p* = 0.0002). Among the residues, *Leucaena* had the highest N content of 3.47%, compared to 1.56% in *Panicum* (Table 1). Regarding P content, only *Leucaena* had a higher content than *Panicum* (*p* = 0.0245). While the legume residues had significantly lower (*p* = 0.0018) LG content than *Panicum*, the latter had a lower PP content than the legumes (*p* = 0.0020). Due to the higher N content in legume residues, estimated quality indicators such as the C:N, LG:N, and (LG + PP):N ratios were substantially lower in legumes compared to *Panicum*. In terms of the PRQI, *Gliricidia* residues scored the highest value of 4.7, indicating superior quality among the tested residues. Other legume residues also had higher quality indices than *Panicum* (2.7 compared to 4.1, 3.5 and 3.3 of *Leucaena*, *Mucuna* and *Pueraria*, respectively).

**Table 1:** Chemical composition of leaf residues and the residue quality index of investigated plant species.

Species	OC (%)	TN (%)	C:N	P (%)	K (%)	PP (%)	LG (%)	LG:N	(LG + PP):N	PRQI
*Leucaena*	46.5 ± 0.2	3.5 ± 0.39**a**	13.4 ± 1.0**c**	0.23 ± 0.05**a**	1.8 ± 0.01**a**	40.6 ± 0.8**ab**	29.8 ± 2.0**cd**	8.6 ± 0.01**d**	20.3 ± 0.5**c**	4.1
*Gliricidia*	46.5 ± 0.2	3.5 ± 0.57**a**	13.5 ± 0.7**c**	0.17 ± 0.00**b**	1.7 ± 0.01**ab**	33.4 ± 3.0**bc**	25.2 ± 2.2**d**	7.3 ± 0.96**d**	17.0 ± 0.5**c**	4.7
*Mucuna*	48.2 ± 0.0	2.9 ± 0.17**b**	16.7 ± 0.2**b**	0.16 ± 0.01**b**	0.9 ± 0.19**c**	47.6 ± 1.6**a**	33.3 ± 3.0**c**	11.5 ± 1.00**c**	28.0 ± 1.5**b**	3.5
*Pueraria*	47.1 ± 0.2	2.5 ± 0.26**c**	18.7 ± 2.0**b**	0.13 ± 0.01**b**	1.5 ± 0.00**b**	26.3 ± 4.1**c**	42.0 ± 2.0**b**	16.7 ± 0.65**b**	27.2 ± 3.2**b**	3.3
*Panicum*	46.4 ± 0.1	1.6 ± 0.41**d**	29.7 ± 1.7**a**	0.12 ± 0.02**b**	1.5 ± 0.22**ab**	17.6 ± 1.9**d**	51.2 ± 0.5**b**	32.8 ± 1.33**b**	44.1 ± 2.9**a**	2.7
**P-value**		**0.0002**	**0.0002**	**0.0245**	**0.007**	**0.002**	**0.0018**	**< 0.0001**	**0.002**	

Values represent means and standard deviation, values in each column not connected by same letter are significantly different.

*Leucaena* *Leucaena leucocephala*, *Gliricidia* *Gliricidia sepium*, *Mucuna* *Mucuna pruriens*, *Pueraria* *Pueraria phaseoloides*, *Panicum* *Panicum maximum.* Note P: available Phosphorus, K: Potassium, PP: Polyphenol, LG: Lignin, OC: Organic Carbon, N: total Nitrogen, C:N : Carbon to Nitrogen ratio, LG:N: Lignin to Nitrogen ratio, (LG + PP):N:Lignin and Polyphenol to Nitrogen ratio, PRQI: Plant Residue Quality Index.

### Soil organic carbon and total nitrogen dynamics and rate of mineralization

Changes in SOC and TN content of treated substrates after plant residue incorporation followed by maize cultivation are presented in Table 2. Amending the substrates with plant residues substantially increased both SOC and TN contents in the treated soils. *Mucuna* residues in the first maize trial contributed the highest amount of SOC (17.3 g kg^− 1^). This represents an approximately 500% increase relative to the initial SOC content (2.9 g kg^− 1^) before amendments application, while un-amended control witnessed about 52% relative gain in SOC. Irrespective of application rate, *Leucaena* residues contributed the highest TN content (2.4 g kg^− 1^), compared to 0.25 g TN kg^− 1^ in the un-amended substrate, resulting in a relative N gain of more than 800%. Combined treatments (*Leucaena* + *Gliricidia*, *Mucuna* + *Pueraria*) resulted in intermediate SOC and TN levels, similar to those of the individual residue with the lower values in each pair.

**Table 2:** Soil organic carbon (SOC) and total nitrogen (TN) dynamics with percentage relative gains in subsoil substrate in response to plant residue incorporation, values represent means and standard deviation.

Soil	SOC (g kg^− 1^)	TN (g kg^− 1^)	C:N ratio	Relative SOC gain (%)	Relative N gain (%)
Un-amended	4.4 ± 0.0	0.55 ± 0.0	8	51.7	120
*Gliricidia*	13.4 ± 3.7	1.70 ± 0.4	7.9	362.1	580
*Leucaena*	16.3 ± 5.0	2.4 ± 0.8	6.8	462.1	860
*Leucaena*+*Gliricidia*	13.3 ± 3.9	1.9 ± 0.9	7.0	358.6	660
*Mucuna*	17.3 ± 4.5	1.8 ± 0.6	9.6	496.6	620
*Pueraria*	13.9 ± 4.5	1.6 ± 0.4	8.7	379.3	540
*Mucuna*+*Pueraria*	15.3 ± 3.8	1.6 ± 0.2	9.6	427.6	540
*Panicum*	15.6 ± 4.9	1.5 ± 0.5	10.4	437.9	500

Soil here represent substrate un-amended or amended with leaf residues of—*Gliricidia*: *Gliricidia sepium*; *Leucaena*: *Leucaena leucocephala*; *Mucuna*: *Mucuna pruriens*; *Pueraria*: *Pueraria phaseoloides*; *Panicum*: *Panicum maximum* and selected combinations. Note: SOC and TN of undisturbed land and mined site were 11.35 and 0.95 g kg^−1^and 2.9 and 0.25 g kg^− 1^, respectively.

The C:N ratios of both un-amended and residue-amended substrates after first maize trial were raised by the amendments compared to the baseline substrate (C:N ratio of 3.8), with *Leucaena*-amended treatments recording the lowest C:N ratio (less than 7), while *Panicum*-amended treatments had the highest C:N ratio (around 10) (Table 2). Correlation analysis (Supplementary Table 1) revealed that TN content in treated subsoil substrates positively correlated with N (*ρ* = 0.7156, *p* = 0.0200) and P (*ρ* = 0.7099, *p* = 0.0215) contents of the residues. Conversely, TN content of the subsoil substrate was negatively correlated with the C:N (*ρ* = −0.7156, *p* = 0.0200), LG:N (*ρ* = −0.6606, *p* = 0.0376), and (LG + PP):N (*ρ* = −0.7340, *p* = 0.0157) ratios of the plant residues.

The rate constant, *k* (per day), representing the mineralization of organic C and N, decreased with increasing rates of residue application (Table 3). Overall, the rate of mineralization in the substrates was very slow. Although *Gliricidia* residues had the highest residue quality index and were expected to exhibit a high mineralization rate for both organic C and N, they recorded the lowest rate constants (0.0009 and 0.0018 per day for C and N, respectively). *Panicum* residues showed the highest rate of N mineralization, a pattern not observed for organic C mineralization (0.0037 per day as the highest N mineralization rate constant, while *Mucuna* revealed the highest C mineralization rate constant of 0.0012 per day).

**Table 3:** Effect of plant residue type and quantity of application on organic carbon and nitrogen mineralization rate constants, *k* (per day) in subsoil substrate.

Residue	C mineralization constant,* k* (per day)/Quantity Applied (t ha^− 1^)			N mineralization constant,* k* (per day)/Quantity Applied (t ha^− 1^)		
	10	20	30	10	20	30
*Leucaena*	0.0014	0.0012	0.0009	0.0030	0.0022	0.0019
*Gliricidia*	0.0012	0.0009	0.0007	0.0025	0.0019	0.0010
*Mucuna*	0.0015	0.0012	0.0008	0.0028	0.0019	0.0016
*Pueraria*	0.0011	0.0009	0.0007	0.0032	0.0019	0.0016
*Panicum*	0.0015	0.0009	0.0009	0.0056	0.0027	0.0027
***k*** **per quantity**	**0.0014**	**0.0010**	**0.0008**	**0.0034**	**0.0021**	**0.0018**

Residue here represent leaf materials of—*Gliricidia*: *Gliricidia sepium*; *Leucaena*: *Leucaena leucocephala*; *Mucuna*: *Mucuna pruriens*; *Pueraria*: *Pueraria phaseoloides*; *Panicum*: *Panicum maximum*, Note: 10, 20 and 30 represent quantities of plant residues applied in t ha^− 1^.

### Effect of residue type and quantity on plant height and SPAD values of maize and cowpea

**Fig. 1:**
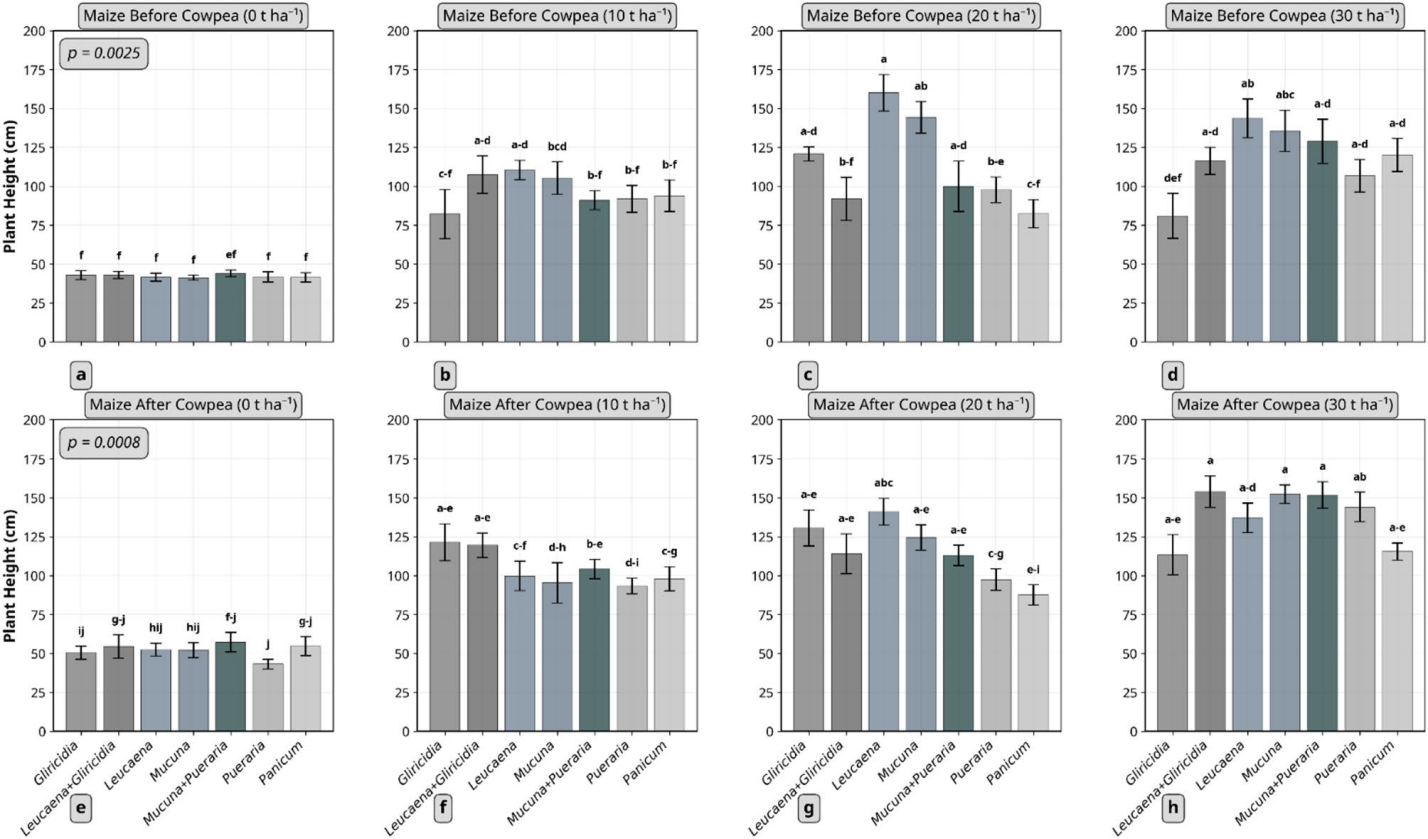
Effect of plant residue type and quantity on maize plant height (8 weeks after sowing). a-d represent maize before cowpea was planted while e-h is the maize after cowpea. Columns not connected by same letter(s) for the interaction between residue type and quantity in each crop sequence are significantly different. Example, mean separation letters a-d denotes the sequence of letters a, b,c, d. Error bars represent standard error. *Gliricidia*: *Gliricidia sepium*; *Leucaena*: *Leucaena leucocephala*; *Mucuna*: *Mucuna pruriens*; *Pueraria*: *Pueraria phaseoloides*; *Panicum*: *Panicum maximum*.

At eight weeks after sowing maize, both residue type and application rate substantially influenced plant height in both trials (maize before and after cowpea) (Fig. 1). Although no significant three way interaction (i.e. crop sequence*residue type*quantity applied) on maize plant height was observed (*p* = 0.4781), significant two way interactions (i.e. crop sequence*residue type, crop sequence*quantity applied and residue type*quantity applied) occurred. Focusing on interactions between residue type and quantity applied within each cropping sequence, significant interaction effects were observed (*p* = 0.0025 and *p* = 0.0008 for the first and second trials, respectively). In the first trial, *Leucaena* residues applied at 20 t ha⁻¹ produced the highest plant height (160 cm), while in the second trial, the *Leucaena* + *Gliricidia* mixture applied at 30 t ha⁻¹ resulted in the tallest plants (154 cm).

For cowpea plant height (Fig. 3a–d) recorded after five weeks of growth, did not reveal any significant interaction between residue type and quantity (*p* = 0.8358). However, there were relatively strong differences among residue application rates, with 30 t ha⁻¹ producing the highest plant height (approximately 50 cm) (*p* < 0.0001) but weak differences between residue type (*p* = 0.0151).

**Fig. 2:**
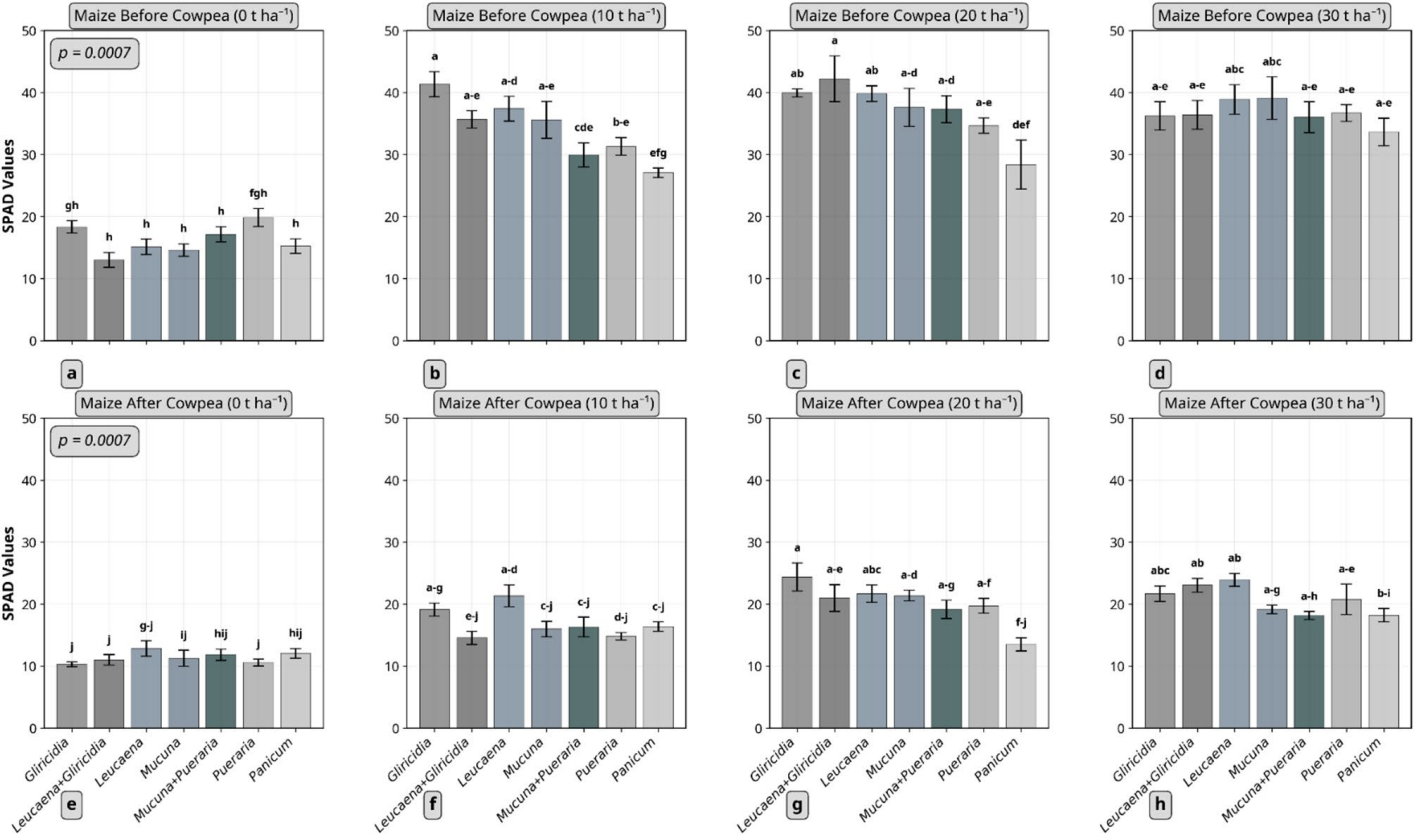
Effect of plant residue type and quantity on SPAD values of maize plants (8 weeks after sowing). a-d represent maize before cowpea was planted while e-h is maize after cowpea. Columns not connected by same letter(s) for the interaction between residue type and quantity in each crop sequence are significantly different. Example, mean separation letters a-d denotes the sequence of letters a, b,c, d. Error bars represent standard error. *Gliricidia*: *Gliricidia sepium*; *Leucaena*: *Leucaena leucocephala*; *Mucuna*: *Mucuna pruriens*; *Pueraria*: *Pueraria phaseoloides*; *Panicum*: *Panicum maximum*.

**Fig. 3:**
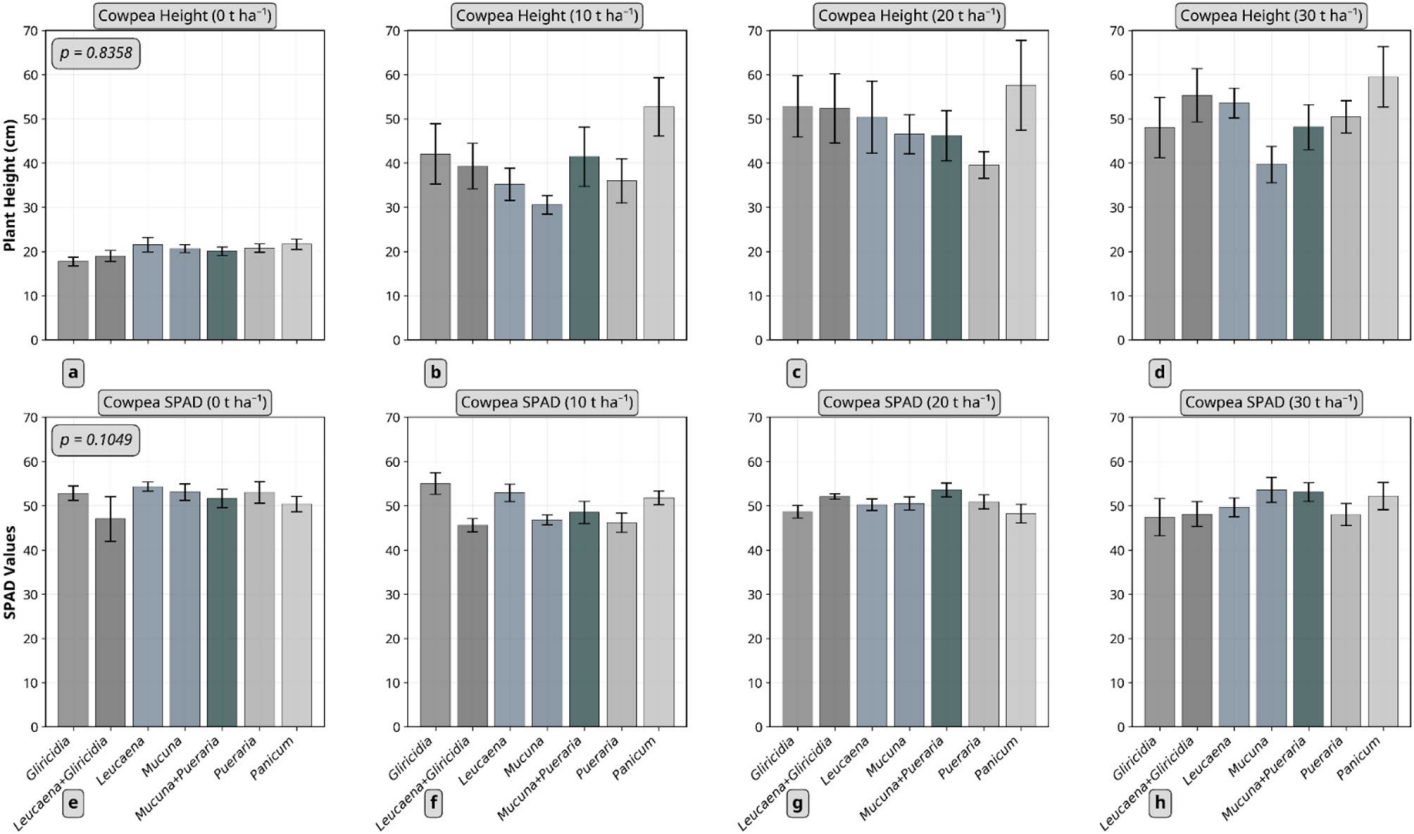
Effect of plant residue type and quantity on the height and SPAD values of cowpea (5 weeks after sowing). a-d represent height while e-h represent SPAD. Error bars represent standard error. *Gliricidia*: *Gliricidia sepium*; *Leucaena*: *Leucaena leucocephala*; *Mucuna*: *Mucuna pruriens*; *Pueraria*: *Pueraria phaseoloides*; *Panicum*: *Panicum maximum*.

Maize SPAD values (Fig. 2) revealed a pattern similar to plant height. There were interaction effects between residue type and quantity in both trials (*p* = 0.0007). In the first trial, the *Leucaena* + *Gliricidia* combination applied at 20 t ha⁻¹ recorded the highest SPAD value (≈ 42), while in the second trial, *Gliricidia* at 20 t ha⁻¹ had the highest SPAD value (≈ 24), indicating a notable decrease between the two trials. For cowpea, no significant interaction effects (*p* = 0.1049) were found for SPAD values (Fig. 3e–h). Further, no significant differences were observed among residue type (*p* = 0.2845) and quantity applied (*p* = 0.3386).

SPAD values obtained from cowpea did not differ between residue-amended treatments and non-amended control, unlike the clear effects seen in maize trials. Additionally, legume residues did not outperform *Panicum* amendments in terms of cowpea plant height or SPAD values, contrasting with the results observed in maize.

### Effect of residue type and quantity on maize aboveground dry matter and cowpea grain yield

**Fig. 4:**
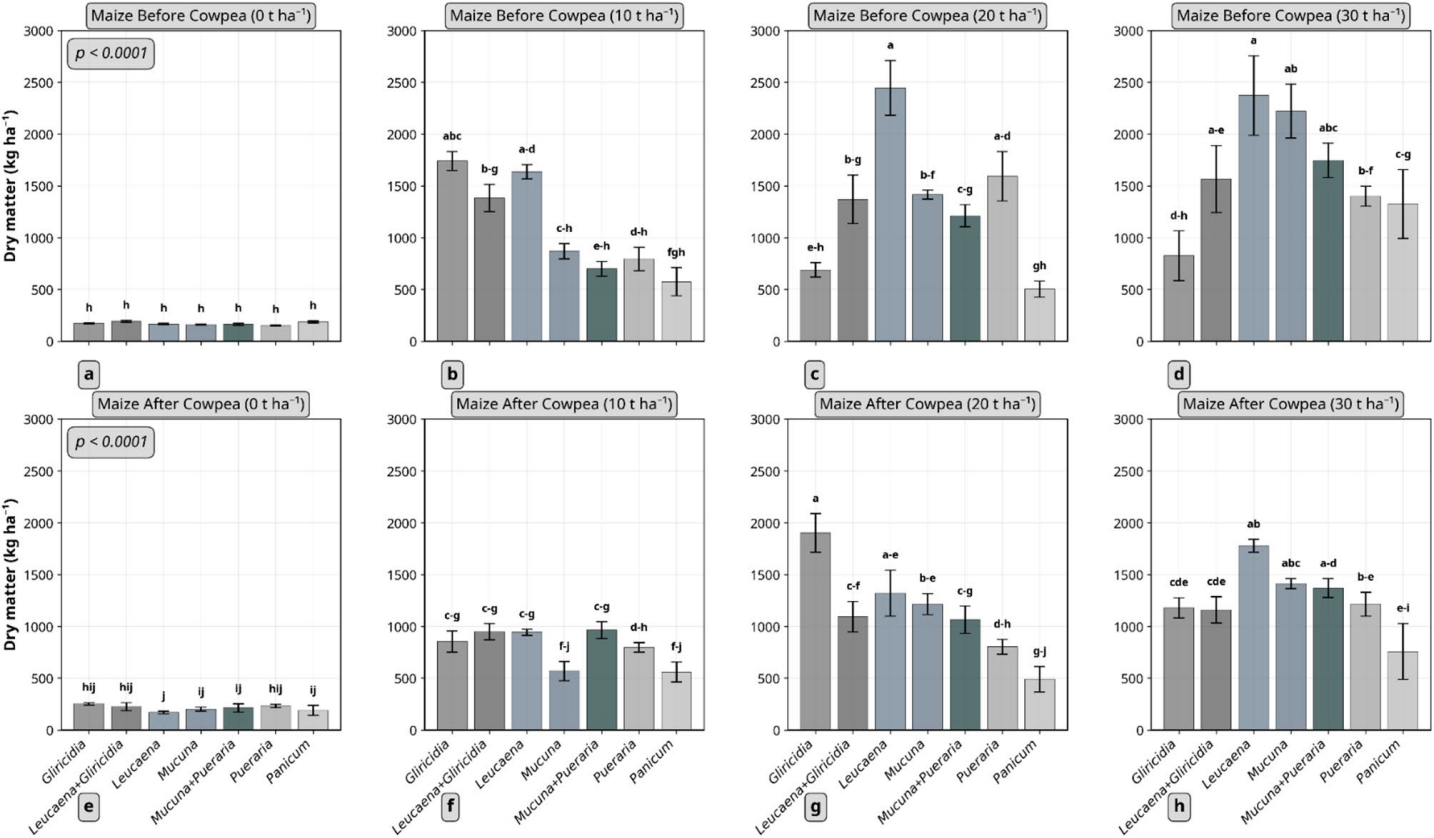
Effect of plant residue type and quantity on maize aboveground dry matter. a-d represent maize before cowpea was planted while e-h is maize after cowpea. Columns not connected by same letter(s) for the interaction between residue type and quantity in each crop sequence are significantly different. Example, mean separation letters a-d denotes the sequence of letters a, b,c, d. Error bars represent standard error. *Gliricidia*: *Gliricidia sepium*; *Leucaena*: *Leucaena leucocephala*; *Mucuna*: *Mucuna pruriens*; *Pueraria*: *Pueraria phaseoloides*; *Panicum*: *Panicum maximum*.

**Fig. 5:**
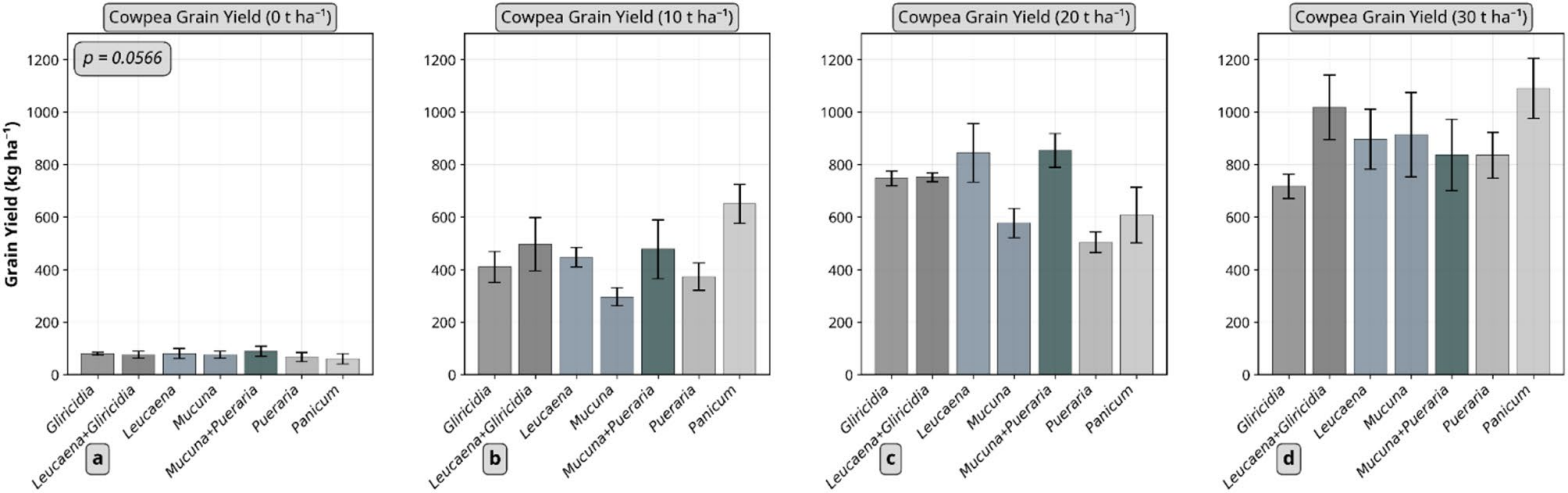
Effect of plant residue type and quantity on cowpea grain yield. Error bars represent standard error. *Gliricidia*: *Gliricidia sepium*; *Leucaena*: *Leucaena leucocephala*; *Mucuna*: *Mucuna pruriens*; *Pueraria*: *Pueraria phaseoloides*; *Panicum*: *Panicum maximum*.

The effects of residue type and application rate on maize dry matter and cowpea grain yield are presented in Figs. 4 and 5, respectively. Both residue type and quantity significantly influenced these crop performance indicators. For maize dry matter, three way interaction effect was observed (*p* < 0.0001). *Leucaena* and *Gliricidia* applied at 20 t ha⁻¹ produced the highest dry matter yields, about 2.4 and 1.9 t ha⁻¹ in the first and second trials, respectively. Legume residues consistently outperformed *Panicum* in both trials. Irrespective of residue type, the highest application rate (30 t ha⁻¹) resulted in the greatest dry matter accumulation (≈ 1.6 and 1.3 t ha⁻¹ in the first and second trials, respectively).

In contrast, maize performance varied substantially in response to the different residue types (≈ 800–2150 kg ha⁻¹ and ≈ 600–1350 kg ha⁻¹ aboveground biomass in first and second maize, respectively) but cowpea did not show such variation (≈ 570–780 kg ha⁻¹ grain yield). Although not statistically different, *Panicum* at 30 t ha⁻¹ produced the highest cowpea grain yield (≈ 1.1 t ha⁻¹), indicating that legume residues did not consistently outperform *Panicum* in the cowpea trial as they did with maize. Dry matter and grain yield in un-amended treatments were very low (< 0.3 t ha⁻¹) compared to residue-amended treatments for both crops.

Maize dry matter in the first trial did not correlate with any residue quality indicators, but several significant correlations were observed in the second trial. Maize dry matter correlated negatively with LG content and C:N ratio (*ρ* = −0.8303, *p* = 0.0029), LG:N (*ρ* = −0.8424, *p* = 0.0022), and (LG + PP):N ratios (*ρ* = −0.8545, *p* = 0.0016). Positive correlations were found with N (*ρ* = 0.8303, *p* = 0.0029) and P content of residues (*ρ* = 0.7095, *p* = 0.0216), as well as with residual soil TN after the first maize trial (*ρ* = 0.8624, *p* = 0.0013). Cowpea grain yield showed positive correlations only with residual SOC (*ρ* = 0.7697, *p* = 0.0092) and TN content (*ρ* = 0.6483, *p* = 0.0426).

## Discussion

### Effect of plant residue quality and quantity on carbon and nitrogen dynamics in subsoil substrate

Legume residues contained markedly more N than *Panicum* residues harvested from the same area. The most plausible explanation is BNF by the legumes soon after their establishment. Although early surveys reported a scarcity of rhizobia able to nodulate *Gliricidia*, Mimosa or Acacia in subsoils^29^, our findings indicate that the introduction of appropriate legume species can rapidly stimulate microbial activity, including rhizobial populations, even in highly degraded substrates. This observation is consistent with reports that revegetation with legumes can rebuild microbial biomass and function in mined soils^14,30–32^. From a practical standpoint, it suggests that, in Ghana and similar contexts where rhizobial inoculants are not widely available^33,34^, direct sowing of selected woody and herbaceous legumes could bypass the inoculation bottleneck and accelerate soil rehabilitation.

Increasing residue load (10 → 20 → 30 t ha⁻¹) consistently slowed the apparent first-order rate constant (k) for C and N mineralization. A likely cause is microbial limitation: the resident decomposer community may be too small to process the larger substrate influx efficiently as the rate of decomposition or degradation is deemed to be proportional to the concentration of decomposers, leading to transient nutrient immobilization^35^. Some studies observed similar trends and attributed this to physical protection through aggregate formation by the higher residue inputs restricting microbial access^36,37^. This finding suggests that applying a single, large dose of high-quality residues can moderate decomposition, potentially synchronizing nutrient release with crop demand, thereby reducing the labor cost of multiple smaller applications in facilitating soil reclamation^15^.

Unexpectedly, *Panicum* residues mineralized N faster than the apparently higher-quality legume residues. Similar discrepancies have been attributed to the protein-binding capacity of PP, which retards N release under non-leaching conditions^38,39^. Because *k* values were derived from residual soil C and N after maize growth, they integrate not only decomposition but also plant uptake and rhizosphere microbial dynamics, further explaining the deviation from chemical-quality predictions. *Gliricidia*, which had the highest PRQI and a chemical profile comparable to *Leucaena*, showed the slowest mineralization. The reduced effect of residue quality at high addition rates concurs with^40^, who reported that increasing residue loads weakened the link between quality indices and decomposition. Correlation analysis confirmed that N release was governed primarily by residue N concentration and the C:N, LG:N and (LG + PP):N ratios. Among these, (LG + PP):N showed the strongest negative correlation with residual soil N, supporting and reinforcing its value as an N-release predictor in legume residues^13^.

### Effect of legume plant residues on productivity of subsoil of degraded mined site

Residue amendment improved crop performance relative to the non-amended control (< 0.3 t ha⁻¹ biomass or grain). Maize responded more strongly than cowpea and revealed pronounced interactions between residue type and application rate. *Leucaena* and *Gliricidia* at 20 t ha⁻¹ maximized maize dry matter (2.4 and 1.9 t ha⁻¹, respectively), whereas 30 t ha⁻¹ sometimes depressed growth, most strikingly with *Gliricidia*. The latter is consistent with transient phytotoxicity reported for *Gliricidia* leachates^41^ and suggests that high-dose surface or shallow incorporation of residues may impede early maize growth until allelochemicals dissipate^42^.

In cowpea, grain yield differences among residue types were minor; *Panicum* at 30 t ha⁻¹ produced the highest yield (≈ 1.1 t ha⁻¹). Because cowpea can meet much of its N requirement through BNF when soil N is low^43–45^ the N advantage of legume residues is less critical. Indeed, mulches with higher N content can suppress BNF^46^, helping to explain why legume residues did not outperform *Panicum*.

Across the sequential trials, performance rankings shifted. Treatments that excelled in the first maize crop declined in the second, indicating residual-nutrient dynamics and possible nutrient stratification. *Leucaena* maintained relatively stable productivity across all three crops, highlighting its potential as a reliable amendment under the studied conditions. Maize failed to set grain in either trial, underscoring the difficulty of restoring heavy-feeding cereals on severely degraded subsoils without prolonged rehabilitation or supplemental fertilizer^47,48^. In contrast, the moderate nutrient demand of cowpea allowed it to produce grain, emphasizing the importance of crop selection during the early rehabilitation phase.

Combined residue treatments (*Leucaena* + *Gliricidia*, *Mucuna* + *Pueraria*) in most instances produced intermediate effects on test crops compared to the individual residues they consisted of. The differences in the chemical composition, rate of decomposition and mineralization of the various organic amendments may have mediated this outcome^49^. Nitrogen-rich organic amendments with low PP and LG content, such as legume residues, are typically readily decomposable^15,50^, leading to mainly short-term effects. Combining legume residues with contrasting biochemical qualities may enhance nutrient balance and provide more sustained benefits, as the results suggest^51^. This may also lead to a more sustained plant growth over relatively long duration through synchronization of nutrient release and plant nutrients requirements at various stages of plant growth and development.

## Conclusions

This study demonstrated that incorporating leaf residues of selected legumes as organic amendments can substantially enhance soil organic carbon and nitrogen levels of subsoil substrates of degraded gold mined sites which is characterized by high SOC and N deficiency. Significant interactions were observed between residue quality (chemical composition), type, and application rate, particularly in influencing test crop performance, indicating species-specific optimal application rates for achieving positive outcomes. Among the evaluated species, *Leucaena leucocephala* produced the most pronounced improvements in soil organic carbon and nitrogen content and crop growth, outperforming the non-legume control *Panicum maximum*. The use of legume residues thus presents a sustainable, environmentally friendly, and cost-effective alternative to conventional organic amendments, while also reducing reliance on topsoil—a scarce resource in mined land restoration. By demonstrating that subsoil productivity can be markedly improved even in the absence of topsoil, this study provides valuable insights applicable to both mined land rehabilitation and organic farming systems. Although based on pot experimentation, the finding establish a critical foundation for developing management and adaption strategies for field implementation.

Prospective field trials are required to validate these results under natural conditions, assess biomass production potential on degraded and marginal lands including farmlands, and optimize management practices such as pruning and cut-and-carry systems. Incorporating these insights into Ghana’s land restoration strategies could play a pivotal role in achieving sustainable recovery of abandoned mined landscapes.

## Supplementary Information

Below is the link to the electronic supplementary material.

Supplementary Material 1

## Data Availability

Data will be made available upon request. For any data request, contact Frank Rasche (the corresponding author).

## Declarations

### Generative artificial intelligence (AI)

During the preparation of this work the authors used ChatGPT in order to improve the readability of the manuscript content. After using this tool, the authors reviewed and edited the content as needed and take full responsibility for the content of the publication.
