# Supplementary material for: Restoring productivity of degraded mined soils using legume leaf residues as organic amendments
Source: Sci Rep. 2026 Mar 6;16:12429. doi: 10.1038/s41598-026-41755-1 (PMC13083890; doi:10.1038/s41598-026-41755-1)
Supplement: Supplementary file 1 — Supplementary Material 1 [file 41598_2026_41755_MOESM1_ESM.pdf]

Restoring productivity of degraded mined soils using legume leaf residues as organic amendments

Enoch Opoku[1], Beloved M. Dzomeku[2], John Opata[3], Adam M. Adam[1], *Frank Rasche[1,4]

Supplementary Table 1 Non parametric Spearman correlation results between residual soil total nitrogen, residual soil organic carbon, cowpea grain yield, maize after cowpea dry matter and some plant residue quality indicators.

Variable	Variable	Spearman coefficient (ρ)	P-value
Residual soil total nitrogen	Lignin+Polyphenol:N of residue	- 0.7340	0.0157
	Total nitrogen content of residue	0.7156	0.0200
	Carbon:Nitrogen of residue	-0.7156	0.0200
	Residual soil organic carbon	0.6973	0.0250
	Lignin:Nitrogen ratio of residue	-0.6606	0.0376
Cowpea grain yield	Residual soil organic carbon	0.7697	0.0092
	Residual soil total nitrogen	0.6483	0.0426
Maize after cowpea dry matter	Lignin content of residue	-0.8303	0.0029
	Total nitrogen content of residue	0.8303	0.0029
	Carbon:Nitrogen of residue	-0.8303	0.0029
	Lignin:Nitrogen of residue	-0.8424	0.0022
	Lignin+Polyphenol:N of residue	-0.8545	0.0016
	Residual soil total nitrogen	0.8624	0.0013

Supplementary Table 2 Random effects covariance parameter estimates report on maize plant height (cm).

Variance Component	Var Ratio	Estimate	Std Error	95% Lower	95% Upper	Wald p-Value	Pct of Total
Rep	0.09365	54.884246	34.967964	-13.6517	123.4202	0.1165	8.555
PotID	0.00105	0.6140082	26.031683	-50.40715	51.635169	0.9812	0.096
Residual		586.04345	47.502649	503.04313	691.54273		91.349
Total		641.5417	54.371726	547.01462	763.01884		100.000

Supplementary Table 3 Fixed effects tests of maize plant height (cm).

Source	Nparm	DFNum	DFDen	F Ratio	Prob > F
CropSequence	1	1	304.4	15.625881	<.0001*
ResidueType	6	6	80.7	8.4852316	<.0001*
Rate	3	3	80.7	243.33778	<.0001*
CropSequence*ResidueType	6	6	304.4	3.0687924	0.0062*
CropSequence*Rate	3	3	304.4	3.1576264	0.0251*
ResidueType*Rate	18	18	80.7	4.8671869	<.0001*
CropSequence*ResidueType*Rate	18	18	304.4	0.983913	0.4781

Supplementary Table 4 Random effects covariance parameter estimates report on maize plant SPAD values.

Variance Component	Var Ratio	Estimate	Std Error	95% Lower	95% Upper	Wald p-Value	Pct of Total
Rep	0.02215	0.5426567	0.4994189	-0.436186	1.5214998	0.2772	2.167
PotID	-0.0469	-1.150313	0.9273772	-2.967939	0.6673128	0.2148	0.000
Residual		24.501736	1.9834304	21.035633	28.906012		97.833
Total		25.044392	2.0366099	21.487144	29.569447		100.000

Supplementary Table 5 Fixed effects tests report on maize plant SPAD values.

Source	Nparm	DFNum	DFDen	F Ratio	Prob > F
CropSequence	1	1	305.2	863.31795	<.0001*
ResidueType	6	6	80.5	13.401847	<.0001*
Rate	3	3	80.5	280.17484	<.0001*
CropSequence*ResidueType	6	6	305.2	1.1846042	0.3142
CropSequence*Rate	3	3	305.2	41.173111	<.0001*
ResidueType*Rate	18	18	80.5	3.5276878	<.0001*
CropSequence*ResidueType*Rate	18	18	305.2	1.5414391	0.0745

Supplementary Table 6 Random effects covariance parameter estimates report on maize aboveground dry matter (t ha^{-1}).

Variance Component	Var Ratio	Estimate	Std Error	95% Lower	95% Upper	Wald p-Value	Pct of Total
Rep	0.05283	4004.3262	4413.8431	-4646.647	12655.3	0.3643	4.946
PotID	0.01526	1157.0344	8477.0105	-15457.6	17771.67	0.8914	1.429
Residual		75796.78	11695.697	57234.81	105169.3		93.625
Total		80958.141	9415.4952	65263.869	103115.24		100.000

Supplementary Table 7 Fixed effects tests report on maize plant aboveground dry matter (t ha^{-1}).

Source	Nparm	DFNum	DFDen	F Ratio	Prob > F
CropSequence	1	1	84.0	30.593777	<.0001*
ResidueType	6	6	81.0	21.623906	<.0001*
Rate	3	3	81.0	215.58578	<.0001*
CropSequence*ResidueType	6	6	84.0	6.2987651	<.0001*
CropSequence*Rate	3	3	84.0	5.8863995	0.0011*
ResidueType*Rate	18	18	81.0	6.6840399	<.0001*
CropSequence*ResidueType*Rate	18	18	84.0	5.3729825	<.0001*

Supplementary Table 8 Effect tests report on cowpea plant height (cm).

Source	Nparm	DF	Sum of Squares	F Ratio	Prob > F
Rep	7	7	1810.180	1.2143	0.2967
Residue Type	6	6	3464.217	2.7111	0.0151*
Quantity Applied (t ha^{-1})	3	3	33258.168	52.0561	<.0001*
Residue Type*Quantity Applied (t ha^{-1})	18	18	2576.687	0.6722	0.8358

Supplementary Table 9 Effect tests report on cowpea SPAD values.

Source	Nparm	DF	Sum of Squares	F Ratio	Prob > F
Rep	7	7	240.8720	0.8144	0.5763
Residue Type	6	6	315.9442	1.2463	0.2845
Quantity Applied (t ha^{-1})	3	3	143.1148	1.1291	0.3386
Residue Type*Quantity Applied (t ha^{-1})	18	18	1117.4394	1.4693	0.1049

Supplementary Table 10 Effect tests report on cowpea grain yield (t ha^{-1}).

Source	Nparm	DF	Sum of Squares	F Ratio	Prob > F
Rep	3	3	80071	1.1164	0.3474
Residue Type	6	6	381872	2.6622	0.0208*
Quantity Applied (t ha^{-1})	3	3	10608043	147.9062	<.0001*
Residue Type*Quantity Applied (t ha^{-1})	18	18	730523	1.6976	0.0566
